# Semen Cryopreservation to Expand Male Fertility in Cancer Patients: Intracase Evaluation of Semen Quality

**DOI:** 10.3390/jpm13121654

**Published:** 2023-11-27

**Authors:** Giuseppina Peluso, Veronica Tisato, Ajay Vikram Singh, Donato Gemmati, Fabio Scarpellini

**Affiliations:** 1 Sperm Bank, Department of Maternal Infant, Annunziata Hospital of Cosenza, 87100 Cosenza, Italy; 2 Department of Translational Medicine, Hemostasis & Thrombosis Centre, University of Ferrara, 44121 Ferrara, Italy; 3 University Strategic Centre for Studies on Gender Medicine, University of Ferrara, 44121 Ferrara, Italy; 4 Department of Chemical and Product Safety, German Federal Institute for Risk Assessment (BfR), 10589 Berlin, Germany; 5 CERM Hungaria, Centre for Reproductive Medicine, 00198 Rome, Italy; ananchekaityche@hotmail.com

**Keywords:** semen cryopreservation, cancer, chemotherapy, epigenetics, fertility risk, fertility preservation

## Abstract

To preserve male fertility after diagnosis of any kind of cancer, a prompt assessment of the semen quality and an appropriate semen cryopreservation must be performed before radio-chemotherapy starts. The present work aims to evaluate the semen parameters at diagnosis of different cancer patients before cryopreservation and after thawing. Testicular tumors and lymphomas are among the most common cancers in younger patients, and while chemotherapy significantly increases patients’ survival, it can epigenetically alter the semen fluid, resulting in temporary or permanent infertility. We analyzed data from the database of the Gamete Cryopreservation Center (Annunziata Hospital, CS; Italy) in the period of 2011–2020 from a cohort of 254 cancer patients aged 18–56 years. The evaluation was performed in a blind manner and anonymously recovered; the main parameters referring to semen quality were assessed in accordance with the WHO guidelines and decision limits (6th edition; 2021). The cancer types were as follows: testis cancers (TC; *n* = 135; 53.1%), hematological cancers (HC; *n* = 76; 29.9%), and other types of cancer (OC; *n* = 43; 17%). Comparing TC vs. HC (P_1_) and vs. OC (P_2_), TC had the worst semen quality: sperm number/mL (P_1_ = 0.0014; P_2_ = 0.004), total motility (P_1_ = 0.02; P_2_ = 0.07), progressive motility (P_1_ = 0.04; P_2_ = 0.05), viability (P_1_ = 0.01; P_2_ = 0.02), and percentage of atypical morphology (P_1_ = 0.05; P_2_ = 0.03). After semen thawing, viability and progressive motility recovery lowered, accounting for 46.82% and 16.75%, respectively, in the whole cohort; similarly, in the subgroups ascribed to TC, they showed the lowest recovery. Strong correlation existed between pre- and post-cryopreservation viability and progressive motility in the whole cohort (*p* < 0.001) and in the TC subgroup (*p* < 0.05). All cancer subgroups, to significantly different extents, had semen findings below the WHO reference values, suggesting diverse sperm susceptibilities to different cancers and cryodamage. Cancer and associated treatments epigenetically affect patients’ semen quality, meaning cryopreservation should be considered a useful personalized prerogative for any kind of cancer in a timely manner.

## 1. Introduction

Cryopreservation of spermatozoa is one of the most valuable and useful strategies to preserve male reproductive function in patients undergoing pharmacological treatments such as chemo- or radiotherapies characterized by a variety of negative side effects. Recently, sperm cryopreservation also has become available for other pathologies or cancers not directly involving the urogenital system, such as autoimmune diseases and blood cancers requiring treatments that may globally affect sperm viability and quality [1]. It has been demonstrated that the rate of successful recovery after semen thawing greatly fluctuates among different pathologies, depending in part on the molecular clusters of somatic mutations detected in the patient responsible for a dysregulation of key proteins associated with sperm fertility and motility [2]. Therefore, sperm parameters assessed early before cryopreservation may predict the post-thawing recovery with high precision [3,4]. Moreover, specialists are more likely to counsel younger female patients on the reproductive risks before initiation of chemotherapies, as recently reported in a large study published on JAMA, and globally less than 45% of patients received counseling concerning the fertility lowering associated with treatments, showing a strong sex gap, with males reaching counseling only 32% of the time [5].

Testicular tumors and lymphomas are among the most frequent malignancies in males during their reproductive age, and thanks to an early diagnosis and the improved efficacy of novel target chemotherapies, the survival rate has significantly increased; however, in the youngest patients, the great psychological problem of treatment-associated infertility remains [6,7,8]. Consequently, the best option to preserve male fertility is semen cryopreservation performed before any antineoplastic treatment, even in presence of abnormal semen parameters, since the intracytoplasmic sperm injection (ICSI) technique allows the use of a single sperm cell to successfully fertilize one egg. Damaging effects of cancer on fertility have been reported [9], and most malignancies, especially Hodgkin’s lymphoma (HL), extragonadal germinoma, and testicular tumors, are often associated with severely altered semen parameters [10,11]. In this regard, several studies documented the negative effects that these forms of neoplasm directly have on spermatogenesis, compromising sperm quality even before any anti-neoplastic treatment starts. Serious histological alterations have been reported in these patients at the level of the epithelium of the seminiferous tubules, probably due to indirect actions on the hypothalamic–pituitary–gonad axis or direct epigenetic effects on the testicular compartment [11,12,13,14,15,16,17,18,19]. However, the specific epigenetics and molecular effects that also other types of tumors have on spermatogenesis remain rather controversial topics [7,8,11,12,13,14,15,16,17,18,19,20,21,22].

In the present study, the main semen parameters of different cancer patients were evaluated before any radio-chemotherapy treatment or cryopreservation and after thawing to assess viability and progressive motility recovery stratified by the different solid and hematological cancers.

## 2. Materials and Methods

### 2.1. Patient Recruitment

Laboratory findings from the database of the Gamete Cryopreservation Center of Annunziata Hospital (CS; Italy) from 2011–2020 were anonymously and retrospectively retrieved, and a total of 254 cancer patients aged 18–56 were analyzed. Cancers were divided into three subgroups according to the specific neoplasia: testicular cancers (TC; *n* = 135), hematological cancers (HC; *n* = 76), and other types of solid cancers (OC; *n* = 43). Data were collected and processed based on the cancer type, age, and quality of the patients’ seminal fluid at the time of cancer diagnosis. Intra-group analyses and computation were performed and the whole data set was compared to the WHO reference indicators. Sperm motility after thawing was assessed for the whole group and the subgroups.

The present study was performed according to the guidelines of the Declaration of Helsinki, and ethical approval was waived since: (i) data were anonymously retrieved and analyzed, (ii) data were obtained during routine clinical visits, (iii) the results did not have any impact on the standard procedure of semen cryopreservation, (iv) the directive staff of the clinic were informed about the ongoing study. Moreover, all the recruited patients signed informed consent to participate in the study.

### 2.2. Semen Quality Assessment

The global evaluation of the seminal parameters included: total number of spermatozoa and number/mL, total and progressive motility, viability, and the percentage of normal morphology in compliance with the WHO 2021 guidelines (https://www.who.int/publications/i/item/9789240030787; accessed on 1 October 2023). Correlations between the seminal parameters and the age of patients stratified by the different types of malignances were also investigated. Pre- and post-cryopreservation mean comparisons and correlations were performed in the whole group and subgroups of cases. Semen samples were collected in a sterile receptacle after 3–5 days of sexual abstinence. After 20–30 min of liquefaction at 37 °C, semen samples were examined for sperm concentration, total sperm count, total and progressive motility (%), viability (%), and morphology (% of typical forms) in a Makler chamber (Sefi Medical Instruments, Ltd. Tel Aviv, Israel). Semen fluid examinations were carried out by the same specialist operator.

#### 2.2.1. Motility Assessment

Sperm motility was evaluated using a phase contrast microscope at 400× magnification. The percentage of motile spermatozoa was evaluated according to WHO guidelines (2021 edition). Basically, four categories of sperm were classified: rapid progressive, slow progressive, non-progressive, and immotile. As a rule, at least 200 spermatozoa in at least five microscope fields were counted for each sample.

#### 2.2.2. Morphology Assessment

Sperm morphology was assessed by smearing 5–10 μL of the sample onto clean glass slides, which was then allowed to air-dry for 20 min at room temperature. Glass slides were stained using smears in the Diff-Quick Kit (Baxter Dade diagnostics AG, Dubingen, Switzerland). Sperm morphology was assessed at 1000· microscope magnification under oil-immersion.

### 2.3. Pre-Freezing and Freezing Procedures

All analyses were carried out on swim-up prepared ejaculate. Fertilization medium (FertiCult ™ Flushing Medium FertiPro; MBT, Italy) was the basic medium for spermatozoa preparation. Briefly, raw ejaculate was diluted 1:1 (*v*/*v*) in the medium and then was pelleted at 400 g × 10 min and the supernatant was discarded. This step was followed by extreme caution; without disturbing the pellet, 0.5–1.0 mL of fresh medium was added and incubated (45 min, 37 °C) according to Meseguer et al., 2006 [23]. After swim-up, sperm suspensions were recovered and rapidly frozen. Samples were initially mixed dropwise in equal volumes of glycerol-based cryoprotectant (Sperm Freezing Medium, Medicult), the most widely used permeating cryoprotectant for human sperm; then, they were gently mixed with continuous shaking at room temperature and maintained at 37 °C for 10–15 min to allow a proper mixing between the cells and the medium. Samples were packaged in frozen paillettes (CBS High Security sperm straw; Cryo Bio System; L’Aigle, France), placed in plastic storage tubes (mini-goblet), and inserted in larger storage goblets. The entire procedure was performed in a class-A classified room (at least D) according to international good manufacturing practice guidelines by utilizing a slow freeze control system consisting of progressive cooling at 1.5 °C/min (from 20 °C to −6 °C), and then at −6 °C/min to −80 °C. Finally, the paillettes were quickly transferred into liquid nitrogen (−196 °C) and stored.

Thawing was carried out by keeping the straws for 15 min at 37 °C in a dedicated dry incubator before further processing.

### 2.4. Statistical Analysis

Statistical analysis was carried out using SPSS (v-22) and GraphPad Prism (v-9.5.0), and all the variables shown were expressed as the mean  ±  SD or percentage. Findings related to sperm quality were analyzed using the Chi-squared test, Student’s *t*-test, or one-way ANOVA. The Pearson correlation test was utilized in the pre- and post-cryopreservation analyses of the whole group and subgroups, and the age-related dynamics of all the semen parameters were centered and scaled according to the formula (x-value − mean value)/SD [Z = (x − μ)/σ]. All the statistical analyses were two-sided, and *p*-values < 0.05 were considered statistically significant.

## 3. Results

### 3.1. Epidemiological Data and Clinical Characteristics

Among a cohort of 254 patients, the most represented cancer was testicular cancer (*n* = 135; 53.1%; among these patients, 92 had seminoma, 68.1%), followed by hematological cancers (*n* = 76; 29.9%; among these patients, 37 had HL, 48.7%) and other types of solid cancers (*n* = 43; 17%; equally distributed among colon, lung, prostate, and bladder).

**Figure 1:**
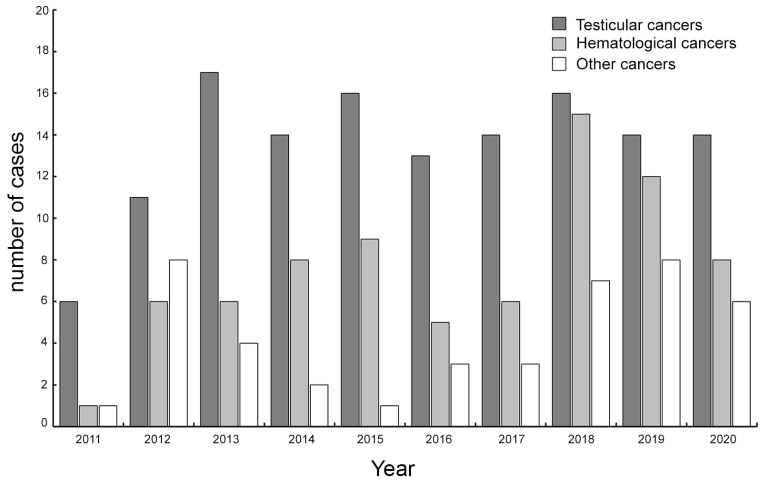
Number of cases recorded from 2011–2020 stratified according to different cancer subgroups.

Figure 1 shows the overall distribution of the entire cohort, depicting the relative frequency of the different cancers from 2011–2020, showing testicular cancer characterized by the highest number of patients compared to the rest of the cancers.

**Figure 2:**
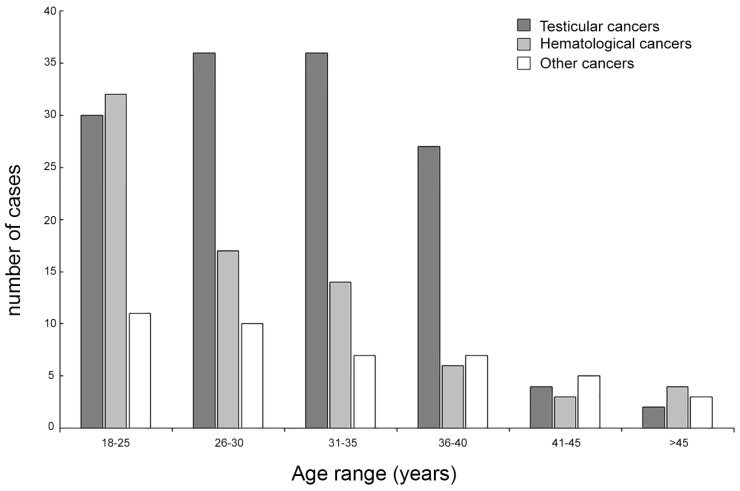
Patient age stratification among the different cancer subgroups.

Figure 2 shows the number of patients stratified according to clusters of age that was significantly overrepresented below 35 years when compared with the remaining clusters of age (*p*-value intra-group comparison, *p* = 0.001), essentially reflecting the reproductive targets of younger males of active reproductive age (75.9% vs. 24.1%; *p* = 0.01).

Approximately 15% of patients used their cryopreserved sperm for ART procedures, 5.5% of patients communicated to the Centre to discard their samples because they did not need cryopreservation anymore, and 6.5% of patients died due to cancer.

### 3.2. Semen Quality Assessment in Cancer Subgroups

Computation and analyses among semen parameters stratified by the various cancer types ascribed the lowest mean number of spermatozoa to testicular cancer patients when compared with the rest of the patients computed together (TC: 15.3 × 10^6^/mL vs. HC + OC: 27.92 × 10^6^/mL; *p* = 0.0005), with a large number of TC cases (70.37%) below the decision limit indicated by the internationally accepted WHO criteria. Therefore, at a greater extent than for other cancer subgroups, the value expressed as percentage of patients below the normal reference number of 15 × 10^6^/mL was significantly overrepresented in testis cancer when compared with the remaining subgroups (Table 1).

We then compared sperm quality findings in the whole group and subgroups, considering the WHO reference values (Table 2). Whilst ejaculate volumes were within the WHO parameters for any group of patients, the number of spermatozoa/mL was below the reference for all the groups, with testis cancers characterized by the lowest number (15.3 mL × 10^6^ ± 23.0) when compared with other cancers (*p* = 0.0014) or hematological cancers (*p* = 0.004). In addition, spermatozoa progressive motility gave the following results: testicular cancer 5.7% ± 7.9; hematological cancers 8.02% ± 9.4 (HL had slight lower motility: 7.02% vs. 8.97%); other cancers 7.1% ± 10.6. This means that testis cancers had the lowest mean value.

Remarkably, morphological analyses expressed as a percentage of the normal phenotype gave mean values above 4% (strict criterion) for all subgroups as follows: testicular cancer 6.59% ± 7.5, hematological cancers 8.75% ± 8.7 (HL had slightly higher rate: 9.54% vs. 8.0%), and other cancers 9.56% ± 9.8, (*p* < 0.01 testis cancers vs. the remaining two subgroups computed together). The wide range of values, extending from 0–35% in hematological cancers and other cancers and from 0–29% in testis cancers, accounts for the high values of SD found in all the subgroups.

As expected by our previous single analyses, testis cancer patients showed anomalous mean values almost for all the quality parameters considered, reaching significant severest mean values or percentages when compared with the other subgroups. Interestingly, cancers not directly affecting the urogenital system also showed abnormal values when compared with the international WHO reference standards, suggesting sperm cryopreservation and preventive biobanking are also useful strategies for these categories of patients.

### 3.3. Comparison and Correlation between Pre- and Post-Cryopreservation Semen Parameters

**Figure 3:**
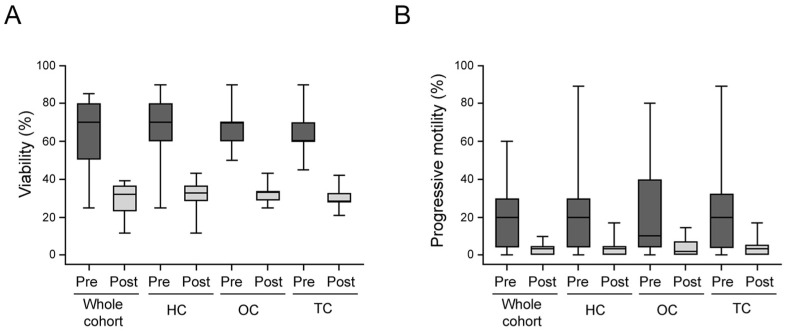
Box plots of the distribution of pre- (Pre) and post-cryopreservation (Post) for viability (**A**) and progressive motility (**B**) stratified by different cancer subgroups. HC, hematological cancers; OC, other cancers; TC, testicular cancer.

Viability and progressive motility were compared in the whole cohort and in subgroups for samples with concentrations ≥2.0 × 10^6^/mL at baseline (Figure 3A,B). Both parameters were significantly lower after thawing in the whole cohort (*p* < 0.001) and in the subgroups (*p* = 0.01). Globally, the whole cohort of cancer patients had a recovery of 46.8% for viability (HC: 47.7%; OC: 46.8%; TC: 45.9%) and of 17.2% for progressive motility (HC: 18.0%; OC: 16.7%; TC: 15.2%). The recovery for the progressive motility of samples with baseline semen concentrations <2.0 × 10^6^/mL was near 0.0% in all the subgroups.

By computing the percentages of pre- and post-cryopreservation viability and progressive motility, significant direct correlations were found in the whole cohort and in the TC subgroup (Table 3); no correlation was found in HC and OC subgroups, probably because of the lower number of cases.

### 3.4. Age-Dependent Impact on Semen Quality

**Figure 4:**
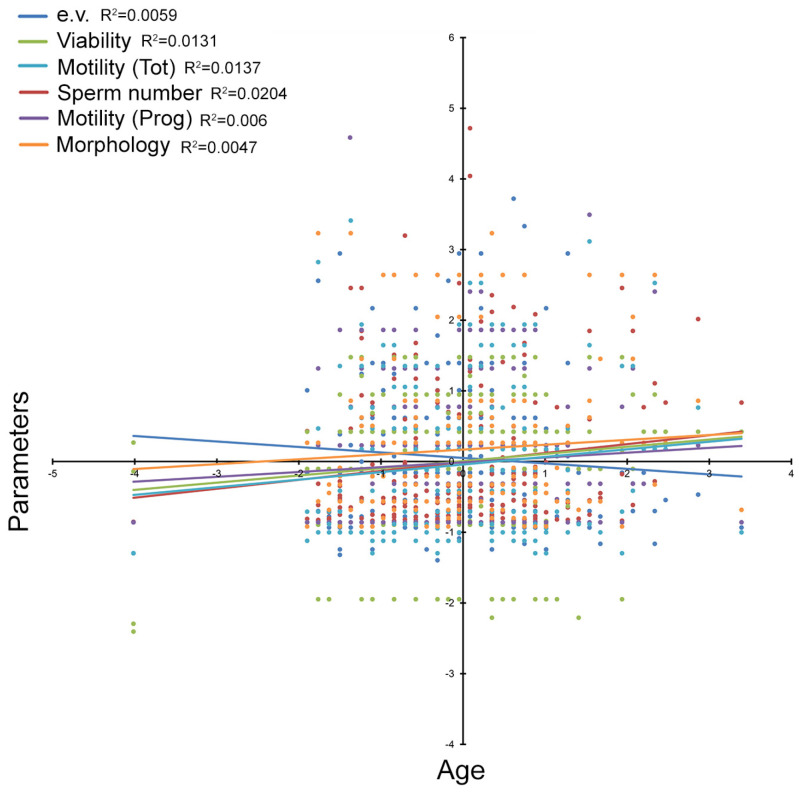
Correlation analyses of the age dependent effects on the sperm quality parameters in the whole cohort of patients. Variables were centered and scaled as described in the Section 2.

To investigate the possible impact of patients’ ages on their semen quality, we correlated sperm parameters with age in the whole group and subgroups. Accordingly, correlation analyses yielded borderline or non-significant results, as summarized in Figure 4 (whole group), which shows trend lines for all the sperm quality parameters and the R^2^-values. In detail, for the whole group, a borderline correlation between age and sperm count was found (R^2^ = 0.0204; *p* = 0.05), together with a non significant inverse correlation between age and ejaculate volume (R^2^ = 0.0059; *p* = n.s.), responsible for a possible combined effect on the final sperm concentration trend. The remaining parameters did not show statistical significance, although, as discussed above, the TC subgroup had the lowest trends for each comparison and parameter considered.

## 4. Discussion

Semen analysis represents a common laboratory basic evaluation of male infertility conditions. Cryopreservation of semen started as clinical practice in the early 1960s and is today performed using different strategies. From the first edition in 1980 to the current sixth edition (https://www.who.int/publications/i/item/9789240030787; accessed on 1 October 2023), there have been important changes and advances in semen examination methods and cryopreservation, improving quality control and assurance [24]. Semen parameter assessment is based on the standards and references listed within the laboratory manual for human semen examination and processing [25,26,27]. Exogenous or endogenous situations can affect sperm quality, and the consequences of cancer and cryopreservation for sperm functions are well known. Spermatozoa can be differently damaged and reflect different freezing–thawing consequences on vital parameters. In this scenario, a personalized semen characterization before cryopreservation stratified by the diverse cancers should be obtained.

The main results observed by the present investigation are that any cancer condition can affect the parameters of semen quality compared to the WHO references, and that this is also reflected in the recovery of functions after sperm thawing regardless of the mean age of patients being comparable among subgroups.

The percentage of patients with sperm counts below the WHO reference value (i.e., ≤15 × 10^6^/mL) was 50% and 56% in HC and OC subgroups, respectively, while 70% of TC patients were below the reference. In detail, TC patients also showed the lowest mean parameters when compared with the other subgroups, particularly for sperm number and total motility, which were about 40% of the respective WHO reference ranges. Conversely, HC and OC retained, respectively, 69% and 75% of the reference concentrations, while comparable mean values were retained for total motility (HC, 51.6%; OC, 45.4%; and TC, 38.1%). Interestingly, progressive motility was severely affected in all the subgroups, which retained low residual motility (HC, 26.7%; OC, 23.6%; and TC, 19.0%) compared to the reference value. Finally, as the mean percentage of viability and morphology is concerned, all the subgroups crossed over the limits of the reference cutoffs.

It has been recently reported that there is a lack of detailed information on motility and viability recovery after semen thawing and whether recovery varies among the different cancers or other pathologies requiring sperm banking [1].

We found that both parameters were significantly lower after thawing in the whole cohort and in the subgroups, globally accounting for 46.8% for viability (HC, 47.7%; OC, 46.8%; and TC, 45.9%) and 17.2% for progressive motility (HC, 18.0%; OC, 16.7%; TC, 15.2%). Additionally, considering a comparable percentage of recovery among the subgroups, particular consideration should be given towards TC patients characterized by halved baseline sperm concentrations compared to HC or OC subgroups, predicting in turn a lower absolute recovery. TC patients are characterized by a direct endogenous exposure of germ cells to the tumor environment enriched by high levels of inflammatory mediators and ROS, and this might greatly affect sperm cells’ global viability due to aberrant epigenomic signals affecting the biological tissue age compared to the classical effects of chronological aging [28,29].

In an explorative attempt, we also investigated whether the considered semen quality findings could be affected by age in the whole group and in the different subgroups. Globally, the ejaculate volume decreased in all subgroups as the age of patients increased, while sperm counts slightly increased, with no significant effects of age observed on the rest of the measured parameters, finally confirming that the TC subgroup had the lowest trends for each comparison and parameter considered.

The main alterations in semen parameters at the time of cancer diagnosis are still being studied by various authors with the aim of improving personalized reproductive chances and preserving male fertility [22,30,31,32]. Various hypotheses have been formulated, and considering testicular tumors, these alterations could be related to the histological type and probably to three main factors: i) biochemical and epigenetic direct damages from the cancer environment to the testicular parenchyma and spermatozoa (also mediated by miRNA and ncRNA); ii) altered endocrine balance; and iii) autoimmunity effects [28,33,34]. As hematological and solid cancer is concerned, the causative alterations in the spermatozoa functions might specifically be due to systemic effects, hypothesized to most likely increase pro-inflammatory circulating cytokines and interleukins in this class of patients [35,36]. Strategies aimed to contrast local oxidative stress and inflammation may positively improve basal- and post-cryopreservation parameters, especially for semen samples with suboptimal features [37,38].

Other non-negligible elements may be related to stressor factors, as a significant psychological component linked to the patient’s awareness of having a cancer disease, which adds to the global semen status already affected by unbalanced individual red-ox homeostasis, particularly at the mitochondria level [39,40,41,42]. As for any cell or tissue involved in complex diseases [43,44,45], in any kind of cancer, different molecular clusters of somatic mutations or inherited predispositions of key genes responsible for the dysregulation of the local redox and metal-homeostasis [46,47] may affect sperm viability and functions, finally disturbing spermatogenesis [2,48,49,50].

Cancer affects the entire body in many negative ways, systemically and locally, and this strongly impairs normal gametogenesis both in males and female. How cancer affects male and female fertility and gametes quality is still partially understood, suggesting the need for multi-OMICs approaches to investigations considering infertility, a complex disease in which males and females show definite features that need to be specifically addressed, and also including genomic and epigenomics investigations [51,52,53,54].

Further multicenter studies including larger case series are needed to better investigate the effects that different neoplastic mutations can exert on sperm cell viability and quality; our results further underline and reinforce the importance of specifically characterizing the basal semen parameters in any cancer to perform early actions and to contrast the modifiable factors in order to preserve and improve fertility scores in these frail patients before any antineoplastic treatments start [31,32,55,56].

Considering that the effects of any malignancy on semen parameters are somewhat debatable, in line with the results reported in the present investigation, it has been established that any type of cancer can significantly impair sperm quality parameters, with effects occasionally still within the normal or borderline range [9].

In addition, one of the most common concerns of patients with cancer, particularly testicular cancer, is the quality of the cryopreserved sperm, which is also affected by individual susceptibility to cryodamage. This may have effects on the types of available assisted reproductive technology (ART), since recent advances in ART have enabled males who were in the past considered infertile to successfully father biological offspring. In this line, the combination of IVF and ICSI has allowed the injection of a single sperm directly into the cytoplasm of one egg. Therefore, unfavorable semen parameters might not affect fertilization or conception rates after appropriate cryopreservation as long as one live sperm can be recovered.

Since the epigenome of spermatozoa can be altered in cancer survivors, primarily after chemo-treatment cycles, and, theoretically, modifications of the sperm epigenome may have possible transgenerational transmission [57,58], it is imperative that oncologists and urologists become more familiar with the several personalized options of fertility preservation and inform patients concerning cancer-associated fertility risks.

Although the data obtained by the present investigation have the limitation of a small number of recruited cases, they are in line with other studies and strengthen the indication that it is beneficial to obtain a personalized semen assessment before treatment and early cryopreservation to avoid lower recovery and potential transgenerational transmission of dangerous epigenomic signatures.

## 5. Conclusions

Cancer, chemotherapy, and other cancer-related treatments have been associated with impairment or loss of future patient fertility, with possible trans-generational transmission. Accordingly, at the time of diagnosis and before the initiation of chemotherapy, detailed counseling explaining the risks must be undertaken. In a study recruiting approximately seven thousand patients, only 45% of cases in reproductive age had a discussion with specialists concerning the risk of infertility associated with treatment [5], a percentage that is too low according to the ASCO recommendations. Globally, there is the need to identify strategies to increase fertility education, and fertility specialists must be involved in counseling at the time of newly diagnosed tumors. Although oncologists recognize the association of chemotherapy and radiotherapy with fertility lowering, they are not well informed on the available fertile gamete preservation options. A stronger cooperation between oncologists, geneticists, and fertility specialists may be of value as a policy to meet the ASCO recommendations.

## Figures and Tables

**Table 1:** Number and percentage of cases with sperm counts below the WHO criteria.

Type of Cancer	≤15 × 10^6^/mL	>15 × 10^6^/mL	*p*-Value *
^a^ Testicular cancer (*n* = 135)	70.37% (95)	29.63% (40)	0.003 (a vs. b)
^b^ Hematol. cancers (*n* = 76)	50.0% (38)	50.0% (38)	0.06 (a vs. c)
^c^ Other cancers (*n* = 43)	55.8% (24)	44.2% (19)	0.0028 (a vs. b + c)

* *p*-value was calculated using Chi-squared, comparing the number in brackets for each subgroup. ^a^, ^b^, and ^c^, indicate Testicular cancer, Hematological cancers, and Other cancers subgroup respectively; a vs. b, a vs. c, and a vs. b + c, indicate the subgroups comparison.

**Table 2:** Semen quality parameters in the whole group and subgroups of cancers.

	Whole Cohort	Hematol. Cancers	Other Cancers	Testicular Cancer	*p*-Value	WHO 2021
Age, mean ± SD	30.2 ± 7.6	28.4 ± 8.5	31.9 ± 9.2	30.7 ± 6.1	0.02 ^a^ n.s. ^b^	- -
Ejaculate volume, mL	2.2 ± 1.3	2.03 ± 1.2	2.48 ± 1.5	2.2 ± 1.3	n.s. ^a^ n.s. ^b^	1.4 (1.3–1.5)
Number/mL, ×10^6^	21.2 ± 28.7	26.9 ± 28.3	29.6 ± 39.9	15.3 ± 23.0	0.0014 ^a^ 0.004 ^b^	39 (35–40)
Total motility, %	18.2 ± 17.8	21.7 ± 18.6	19.1 ± 20	16.0 ± 16.3	0.02 ^a^ 0.07 ^b^	42 (40–43)
Progressive motility, %	6.6 ± 8.9	8.02 ± 9.4	7.1 ± 10.6	5.7 ± 7.9	0.04 ^a^ 0.05 ^b^	30 (29–31)
Viability, %	62.3 ± 18.7	67.1 ± 16.3	59.4 ± 18.4	60.3 ± 19.9	0.01 ^a^ n.s. ^b^	54 (50–56)
Morphology, %	7.73 ± 8.4	8.75 ± 8.7	9.56 ± 9.8	6.59 ± 7.5	0.05 ^a^ 0.03 ^b^	4 (3.9–4)

The *p*-values were calculated by comparing testis cancers with hematological cancers (^a^), and other cancer (^b^) subgroups, respectively.

**Table 3:** Correlation between viability and progressive motility pre- and post-cryopreservation.

Pre-Cryopreservation				
Post-Cryopreservation			**Viability (%)**	**Progressive Motility (%)**
	Whole cohort	Viability, %	R^2^ = 0.35; *p* < 0.001	R^2^ = 0.42; *p* < 0.001
		Progressive motility, %	R^2^ = 0.35; *p* < 0.001	R^2^ = 0.45; *p* < 0.001
	Hematological cancers (HC)	Viability, %	R^2^ = 0.12; *p* = n.s.	R^2^ = 0.16; *p* = n.s.
		Progressive motility, %	R^2^ = 0.18; *p* = n.s.	R^2^ = 0.13; *p* = n.s.
	Other cancers (OC)	Viability, %	R^2^ = 0.09; *p* = n.s	R^2^ = 0.06; *p* = n.s
		Progressive motility, %	R^2^ = 0.08; *p* = n.s	R^2^ = 0.07; *p* = n.s
	Testis cancer (TC)	Viability, %	R^2^ = 0.2; *p* < 0.05	R^2^ = 0.18; *p* < 0.05
		Progressive motility, %	R^2^ = 0.16; *p* < 0.05	R^2^ = 0.2; *p* < 0.05

## Data Availability

The original contributions presented in the study are included in the article.
